# Neuromodulation of Attention

**DOI:** 10.1016/j.neuron.2018.01.008

**Published:** 2018-02-21

**Authors:** Alexander Thiele, Mark A. Bellgrove

**Affiliations:** 1 Institute of Neuroscience, Newcastle University, Newcastle upon Tyne, UK; 2 Monash Institute of Cognitive and Clinical Neurosciences (MICCN) and School of Psychological Sciences, Monash University, Melbourne, Australia

**Keywords:** attention, pharmacology, top-down, neuromodulators, attractor networks, population coding

## Abstract

Attention is critical to high-level cognition and attention deficits are a hallmark of neurologic and neuropsychiatric disorders. Although years of research indicates that distinct neuromodulators influence attentional control, a mechanistic account that traverses levels of analysis (cells, circuits, behavior) is missing. However, such an account is critical to guide the development of next-generation pharmacotherapies aimed at forestalling or remediating the global burden associated with disorders of attention. Here, we summarize current neuroscientific understanding of how attention affects single neurons and networks of neurons. We then review key results that have informed our understanding of how neuromodulation shapes these neuron and network properties and thereby enables the appropriate allocation of attention to relevant external or internal events. Finally, we highlight areas where we believe hypotheses can be formulated and tackled experimentally in the near future, thereby critically increasing our mechanistic understanding of how attention is implemented at the cellular and network levels.

## Teaser

Attention is a critical function that sculpts neuronal processing to benefit perception, decision making, and action. This review by Thiele and Bellgrove provides a state-of-the-art perspective on the contributions of different neuromodulators to the effects of attention on neuronal activity.

## Main Text

The limited processing capacity of the perceptual system poses a complex computational problem for humans and other organisms: which inputs are relevant to current behavioral goals? Decades of research has now been devoted to understanding how neurons instantiate the required “selectivity” that allows an organism to prioritize, or bias, the processing of relevant over irrelevant inputs. Neuronal processing may be biased by both bottom-up and top-down influences. The former reflects the biasing of sensory processing due to stimulus saliency (brightness, movement, size, for example), which causes features to “pop-out” from their surroundings to capture attention. Top-down processing on the other hand reflects the voluntary guidance of attention to locations, features, or objects in the environment. In this way, top-down attention allows for the voluntary processing of relevant over irrelevant inputs in line with the current behavioral goals of the organism (Desimone and Duncan, 1995).

A network of prefrontal and parietal cortical areas is critically involved in the selection required for top-down attention, and other high-level cognitive functions such as working memory or inhibitory control (Bichot et al., 2015, Corbetta and Shulman, 2002, Fedorenko et al., 2013, Moore and Armstrong, 2003). The state and functionality of this network depend on tightly controlled activity in brainstem neurons that release neuromodulators at their target sites. Neuromodulators configure neuronal circuits and thereby specify output properties (Marder, 2012). They thus shape information processing in local and large-scale neuronal networks, such that relevant, over irrelevant, information is prioritized. This in turn drives behavior the subject hopes to be rewarding or behavior that minimizes adverse outcomes. Understanding precisely how this is achieved at the level of single neurons, local networks, or large-scale networks is vital for basic and clinical neuroscience. Neuromodulators most strongly implicated in high-level cognitive functions are acetylcholine (ACh), dopamine (DA), noradrenaline (NA), and serotonin (5-HT). In this review, we focus on their relevance for top-down attentional control. While we will focus on their role in relation to attention, the above neuromodulators have major roles in other aspects of cognition, such as reward signaling (e.g., ACh and DA, Richardson and DeLong, 1986, Rutledge et al., 2015, Schultz, 2015), working memory (DA, NA, Arnsten and Goldman-Rakic, 1990, Gamo et al., 2010, Mao et al., 1999, Sawaguchi and Goldman-Rakic, 1994, Wang et al., 2004, Williams and Goldman-Rakic, 1995), or inhibitory control (NA, DA, 5-HT, Chamberlain et al., 2006, Nandam et al., 2011, Nandam et al., 2013, Nandam et al., 2014, Winstanley et al., 2006).

An important point to address from the outset is how attention, i.e., the top-down prioritization of behaviorally relevant inputs, differs from working memory. Working memory can be conceived as an active process whereby stimulus or internal representations are stored “on-line” to prevent temporal decay or intrusion from competing or distracting stimuli that are outside the current focus of attention. Dissociating effects of attention from those of working memory is difficult and in practice the two processes are highly interactive (Awh and Jonides, 2001). Attention, as conceptualized in this paper, is a selection mechanism that allows for the preferential processing of task-relevant information over irrelevant (distracting) information, i.e., it is a filter mechanism. This selection is driven by currently active behavioral goals held in working memory. In that sense, attention acts in the service of working memory. However, behavioral paradigms employed in neurophysiological studies highlight the potentially close coupling of attention and working memory (e.g., cue-guided spatial working memory tasks or cue-guided spatial attention task). In the cue-guided spatial working memory task, a brief spatial cue will capture attention, which then triggers the working memory signal that enacts the behavioral goal of a memory-guided saccade to cued spatial location. We contend that covert spatial attention would also be allocated to the cued location during the memory period, rendering the two processes largely inseparable. Here, the neural correlates of either process would be “delay activity”; that is, activity that occurs (or persists) even after a behaviorally relevant stimulus (or cue) is removed from the sensory environment (Fuster, 1973). Similarly, in a cue-guided spatial attention task, a transient cue (either spatial or symbolic) will activate a spatial working memory signal specifying a location that should be monitored for the occurrence of a target, and this monitoring (the preferential processing of information) will be performed by spatial selective attention. Again, in this scenario the effects of attention and working memory during the monitoring period are difficult to dissociate, even if the initial generation of the top-down goal (the working memory) may be briefly separable from the attentional signal (the monitoring itself). Given the pervasive use of these behavioral paradigms in monkey neurophysiology (e.g., Chang et al., 2012, Funahashi et al., 1989), we draw on evidence from both in this review.

The focus of this review will not be on how the different receptors affect specific aspects of cellular signaling, or how behavioral studies have informed our knowledge of cognitive aspects of attention. Rather, we aim to delineate how these neuromodulators enable attentional signaling, either through direct action, or by enabling network states, which favor top-down attentional selection. Such a low-level mechanistic account is necessary to validate work that conceptualizes high-level neuromodulator functions from a computational and theoretical perspective (for example, Yu and Dayan, 2005), and it may help to understand why neuropharmacological manipulations can be task and context specific. Finally, such a mechanistic account is required to guide the development of next-generation pharmacotherapies. We note from the outset that, although a full understanding of the neuromodulation of attention is not possible given current data, we review the state-of-the-art and highlight important questions that can be addressed in the near future.

Neuromodulators act mostly through metabotropic receptors, which activate different G-proteins and thereby trigger second messenger cascades. They affect different receptor types and can thereby enhance or reduce transmitter release, synaptic efficacy, or postsynaptic excitability in neuronal circuits. Exceptions to the metabotropic neuromodulator pathways exist for ACh, which can act through ionotropic nicotinic receptors, and for 5-HT, which can act through the ionotropic 5-HT3 receptor. Detailed reviews regarding the different receptors involved and their action can be found elsewhere (e.g., Beaulieu and Gainetdinov, 2011, Hein, 2006, Nichols and Nichols, 2008, Thiele, 2013). The effects of these neuromodulators follow the classic Yerkes-Dodson (inverted U-curve) dose-response relationship (Yerkes and Dodson, 1908). This describes the phenomenon that either too little or too much neuromodulatory drive is equally detrimental to cognitive ability. This relationship has been described for ACh (Smucny et al., 2015), DA (Vijayraghavan et al., 2007), NA (Aston-Jones and Cohen, 2005, Aston-Jones et al., 1999), and 5-HT (Cano-Colino et al., 2014).

To link neuromodulatory action to attentional effects on neuronal activity, we first provide a brief account of how top-down attention affects neuronal activity, i.e., attention that is directed to spatial locations, objects, or features of the world. Detailed review articles of these effects can be found elsewhere (e.g., Desimone and Duncan, 1995, Maunsell and Treue, 2006, Maunsell, 2015, Miller and Buschman, 2013). Our aim here is to link the effects of attention on single neurons and circuits to those same effects reported for neuromodulators. This link is in many cases indirect, as our knowledge of the actions of neuromodulators on cell and population activity arises largely from studies where attention was not manipulated. Nevertheless, the extant data allow us to delineate likely scenarios that describe how neuromodulator actions could aid attentional selection.

### Effects of Attention on Neuronal Processing

Over the course of the past 30 years, a large number of studies have delineated the neuronal signatures of top-down attention. Top-down attention is defined as the cognitive process by which an individual selects and prioritizes the processing of specific aspects of information. This cognitive process has neural correlates, and we use the term “top-down attention” as a descriptor of neuronal activity changes that occur following the selection process. The behavioral benefits associated with top-down attentional selection are then an emergent property of activity changes in single neurons and in neuronal populations that occur within an area and between multiple areas.

**Figure 1:**
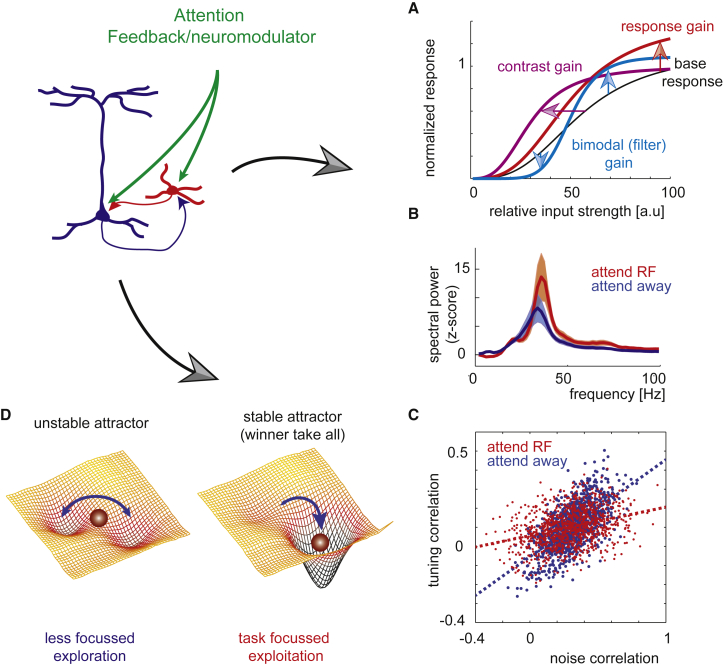
Schematic that Exemplifies Some of the Effects Attention Has on Cellular Activity and on Population Activity Feedback and neuromodulator influences alter the drive in excitatory (blue) and in inhibitory (red) cells, which thereby exert increased influence on one another and the rest of the network, leading to overall increased, but balanced excitation, and inhibition. Exemplified are three scenarios, which have been described in the literature when attention is deployed to a spatial location and/or to specific stimulus features. These can also occur when neuromodulators are applied to the local network. (A) A result thereof is a gain change, i.e., a change of the output a neuron produces given a specific input. (B) Another effect of increased balanced excitation and inhibition is an increase in gamma oscillations of the local field potential, indicative of higher neuronal coherence, proposed to improve information exchange between selected neuronal populations. (C) Attention and neuromodulators also change the relationship between tuning similarity and noise correlations of neuron pairs, such that population coding properties are improved. (D) These changes are reminiscent of altered network attractor dynamics, which stabilize network states, reduce fluctuations, and increase the ability to stay task focused. a.u., arbitrary units; Hz, frequency in Hertz.

One of the main neuronal correlates of attention is an alteration to neuronal firing rates (Reynolds et al., 2000, Spitzer et al., 1988, Treue and Maunsell, 1996). Neurons that represent the focus of attention respond differently to the inputs they receive. In general, neurons representing attended locations, features, or objects show increased firing rates. This phenomenon has been observed across all cortical and subcortical areas investigated (e.g., Krauzlis et al., 2013, Noudoost et al., 2010). However, attention can also result in suppression of neuronal activity for unattended locations or features (Martinez-Trujillo and Treue, 2004). The ways in which attention changes neuronal input-output relationships were originally captured by different models of “gain change.” Gain change describes how neuronal input-output relationships are affected by attention. These gain changes can take different forms, such that responses are either proportionally increased for all stimuli (response gain change), or mostly for less-salient stimuli (contrast gain change), or by a reduction in responses to less-salient (non-preferred) stimuli and an increase in responses to salient (preferred) stimuli (see Figure 1A, Martinez-Trujillo and Treue, 2004, McAdams and Maunsell, 1999, Reynolds et al., 2000, Thiele et al., 2009, Williford and Maunsell, 2006). These different models can, to some extent, be unified within the normalization model of attention (Lee and Maunsell, 2009, Mitchell et al., 2009, Ni et al., 2012, Sanayei et al., 2015). This model assumes that attention affects the gain of excitatory, but also of inhibitory neurons, thereby normalizing the increased excitation induced by attention. In extrastriate and frontal cortex, attention does indeed increase the activity in putative excitatory and putative inhibitory cells (Mitchell et al., 2007, Thiele et al., 2016). At first glance, it may seem counterintuitive that attention increases firing rates, when increased inhibition should counter such an effect. However, this effect can be understood in terms of competing neuronal populations that represent different locations of features (Reynolds et al., 1999). Attention alters the balance between excitation and inhibition of this competition (Reynolds and Heeger, 2009). The attention-induced normalization supports neuronal computations that enable winner-take-all states (Carandini and Heeger, 2011). Winner-take-all network states occur, for example, when attention biases one neuronal population over another to win the competition for sensory representation (Reynolds et al., 1999, Wang, 2008). The winning population shows increased neuronal activity and suppresses its competitors. Competition can also occur in the decision space. When multiple decisions are possible but competing, the interaction between the representations converges toward a single (winning) choice (Reynolds et al., 1999, Wang, 2008). These winner-take-all states occur naturally in neuronal attractor networks. Attractor networks move from labile states, where they can undergo regular and easy state transitions, to stable (attractor) states, where one representation dominates. The stability of these states is determined by the strength of inhibitory and excitatory drive (Deco and Hugues, 2012, Deco et al., 2014). By increasing inhibitory and excitatory drive, attention can allow competing unstable patterns of neuronal activity (representations) to converge to a more stable pattern of activity, such that a single representation dominates (see Figure 1D for a schematic). These stable activity patterns can be associated with a specific attentional state (such as a location), an item held in working memory, a decision that has been formed, or a motor plan. Importantly, the stability of the activity pattern in the attractor network (illustrated by the depth of the valley in Figure 1D) can be influenced by different neuromodulators (see later parts of the review). Cognitive operations or neuromodulators supporting labile states would enhance cognitive flexibility (but potentially at the cost of increased distractibility), whereas cognitive operations or neuromodulators supporting stable states would benefit task focus.

Stabilized attractor networks are characterized by increased firing rates for the winning population and decreased firing rates for the losing populations (the described gain changes). In addition, stabilized attractor networks exhibit reduced neuronal rate variability and rate co-variability (Deco and Hugues, 2012, Deco et al., 2014). These effects equally occur in neuronal populations representing the focus of top-down attention (Cohen and Maunsell, 2009, Herrero et al., 2013, Mitchell et al., 2007, Mitchell et al., 2009, Rabinowitz et al., 2015, Ruff and Cohen, 2014, Thiele et al., 2016). Increased neuronal gain and reduced rate variability both increase the signal-to-noise ratio (SNR), that is, the ability of neurons (or neuronal populations) to distinguish between a relevant and an irrelevant stimulus, to detect the presence or the absence of a stimulus, or discriminate between two different stimuli. Alteration of rate co-variability (also termed noise correlation) determines to what extent the neuronal activity in any two cells is redundant and thus limits how much additional information can be obtained by decoding the input from both neurons compared to just one. High levels of co-variation can alternately be detrimental, beneficial, or irrelevant (Abbott and Dayan, 1999, Panzeri et al., 1999). These outcomes are determined by the tuning similarity between the neurons. Specifically, a certain change to the correlational structure of neuronal tuning and of the rate co-variability (noise correlations) increases the amount of information neuronal populations can encode, largely by reducing redundancy (see Figure 1C for a schematic of this scenario). Thus, attention induces gain change, reduces rate variability, and alters noise correlations, which jointly can increase population coding abilities, i.e., the amount of information a population of neurons can encode about different stimulus or task conditions. Importantly, altered neuromodulator drive can affect neuronal gain, rate variability, and noise correlations in ways similar to attention.

The increase in inhibitory drive may also contribute to attention-induced changes in oscillatory activity in the gamma frequency range (Figure 1B), which requires cyclic bouts of excitation-inhibition (Buschman and Miller, 2009, Chalk et al., 2010, Fries et al., 2001, Gregoriou et al., 2009, Kohl and Paulsen, 2010). Increased oscillatory activity may improve communication between selected neurons within and between brain areas (Bosman et al., 2012, Gregoriou et al., 2009). However, attention does not solely affect the strength of gamma frequency oscillations. Changes in lower-frequency bands, such as theta (Chang et al., 2016, Womelsdorf et al., 2010), alpha (Bauer et al., 2012, Bonnefond and Jensen, 2015, Buffalo et al., 2011, Buschman et al., 2012, Deco and Thiele, 2009, Jensen et al., 2014), and beta frequency (Bauer et al., 2012) equally are prominent and are assumed to attain specific functional roles. For example, desynchronized alpha oscillations are generally associated with enhanced top-down attentional control (O’Connell et al., 2009), and beta band oscillations have been discussed as a mechanism that promotes feedback influences (Fries, 2015).

It is well established that frontal and parietal areas control top-down attention (Corbetta and Shulman, 2002). These areas induce attentional effects in sensory and associative areas through feedback connections (Buschman and Miller, 2009, Gregoriou et al., 2009, Moore and Armstrong, 2003). In addition, frontal areas are connected to brainstem neuromodulator nuclei, whereby they affect their own neuromodulator tone, as well as the neuromodulator tone in other brain areas (Dembrow and Johnston, 2014). This pattern of connectivity may account for the similarity of neuronal changes associated with top-down attention and those seen when the brain undergoes large-scale “state” changes that are under neuromodulator control (Harris and Thiele, 2011). Because of this, it has been argued that the changes associated with attention are linked to large-scale changes but occur at a more local level and show more modest effects (Harris and Thiele, 2011). Attention indeed controls cortical state in circumscribed neuronal populations within task relevant brain regions (Engel et al., 2016, Rabinowitz et al., 2015). Whether these state changes are induced by direct feedback, by alteration of neuromodulator tone, or by a mix of the two is unknown. An equally important question is whether neuromodulators, through tonic release, simply enable network states upon which feedback input can act to induce the described attentional effects. Alternatively, or in addition, neuromodulators play a more active role, whereby their phasic and more local release directly supports states of attention at the neuronal level.

In the sections that follow, we review the evidence for a direct role of distinct neuromodulators (ACh, DA, NA, 5HT) in attention. This will be followed by more indirect arguments, where the action of a neuromodulator at the cellular or network level recapitulates the action of attention, but for which direct evidence linking the two is not available.

### ACh and Attention

The cortically projecting cholinergic system, arising in the basal forebrain (BF), has long been associated with different cognitive functions, such as arousal, attention, learning, memory, and even consciousness (Everitt and Robbins, 1997, Hasselmo and Sarter, 2011, Sarter and Bruno, 1997). However, its contribution to attention has been challenged, on the basis that BF cholinergic projections to the cortex lack spatial specificity (Mesulam et al., 1986, Sarter et al., 2009) and temporal precision (Sarter et al., 2009) and are strongly involved in global brain state changes (Lee and Dan, 2012, Moran et al., 2013, Varela, 2014), such as transitions from sleep to wakefulness, or from quiet wakefulness to active exploration (Harris and Thiele, 2011, Vanderwolf, 1988). These enduring states are assumed to be driven by changes in tonic and burst (phasic) activity levels of BF cholinergic neurons (Lee et al., 2005). In contrast, top-down attention is a mechanism that has high selectivity in the spatial, feature, or object domains, and it can act with high temporal precision (Coull and Nobre, 1998). Yet, ample evidence points to a specific involvement of the cholinergic system in top-down attention. For example, lesions to the cholinergic system in monkeys and rodents produce selective attentional deficits, while sparing other cognitive functions such as learning and memory (Dalley et al., 2004, Gill et al., 2000, McGaughy et al., 1996, Voytko et al., 1994). Increased cholinergic drive benefits attentional performance particularly under high task demand and in the presence of distracting stimuli (St Peters et al., 2011). Finally, polymorphisms of the gene (*CHRNA4*) encoding the α(4) subunit of α(4)β(2) nicotinic receptors have been associated with individual differences in top-down attentional control (Greenwood et al., 2009).

**Figure 2:**
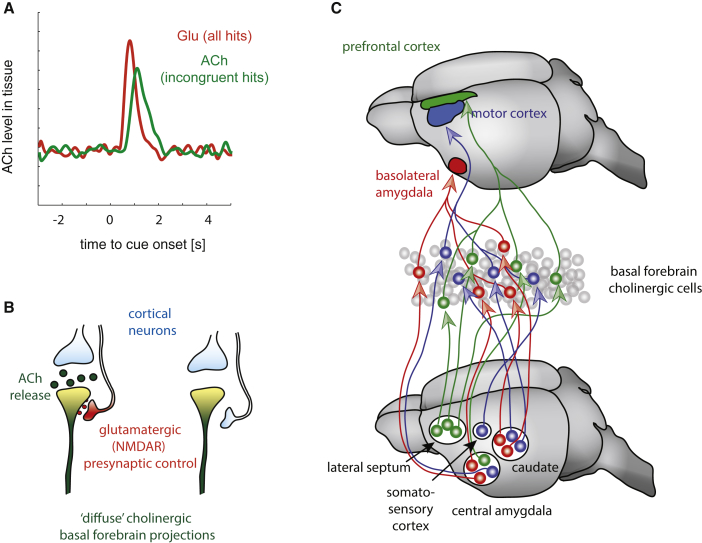
Influence of Tonic and Phasic Modes of Cholinergic Signaling on Attention, and Spatial Specificity of Cholinergic Input-Output Relations (A) Tonic levels of ACh in rodent prefrontal cortex are interspersed by brief phasic increases in ACh, which occur after behaviorally relevant cues, but only if they occur after “non-cue” trials. These are preceded by glutamate increases, which occur on all “cue detect” trials (after Sarter et al., 2014). (B) Potential source of spatial and temporal specificity of ACh signals in the cortex. ACh release in rodent prefrontal cortex is partly dependent on local glutamate activating presynaptic NMDA receptors. Local glutamate control of ACh in rat prefrontal cortex is released from mediodorsal thalamic inputs. Whether other glutamate sources (e.g., feedback from higher cortical areas) equally control ACh release locally is unknown. (C) Input to and output from cholinergic basal forebrain neuron is segregated into specific sub-circuits. Cholinergic BF neurons that project to the prefrontal cortex receive input mostly from neurons in the lateral septum, and from small, but segregated populations of the central amygdala. Cholinergic BF neurons that project to the motor cortex receive inputs from neurons in the somatosensory cortex, and segregated populations of neurons in the central amygdala and the caudate nucleus. Cholinergic BF neurons that project to the basolateral amygdala receive input from segregated populations in the central amygdala and the caudate nucleus (after Gielow and Zaborszky, 2017).

In addition, the spatial specificity of BF cholinergic projections is much more precise than previously thought (Gielow and Zaborszky, 2017, Zaborszky et al., 2015), whereby cholinergic neurons can project to fairly localized parts of the cortex (or subcortical areas, Figure 2C for a cartoon) and in turn have highly selective input relationships (Gielow and Zaborszky, 2017).

Activity in cholinergic neurons is not only related to global behavioral states (Lee et al., 2005). Although cholinergic neurons show tonic activity that is state dependent (Manns et al., 2000), they also generate temporally precise phasic ACh release in rat prefrontal cortex upon attention demanding cue detection (Figure 2A; Parikh et al., 2007, Sarter et al., 2009). Local glutamate co-release is necessary, but not sufficient for these transients to occur (Sarter et al., 2014). Importantly, precisely timed activation of cholinergic neurons by means of channelrhodopsin increases cue detection probability and causes increased levels of false alarms on no-cue trials. Finally, precisely timed inhibition of these neurons results in reduced likelihood of cue detection (Gritton et al., 2016). Moreover, optogenetic activation of cholinergic BF neurons, or their terminals in V1, increases trial-by-trial performance in mice in a visual detection task (Pinto et al., 2013).

How ACh enables neurons to generate attention-related signals is poorly understood. Attention-induced rate changes in macaque primary visual cortex (V1) depend on muscarinic, not nicotinic ACh receptors (Herrero et al., 2008). At the same time, behavioral studies imply a role of muscarinic and nicotinic receptors in attention (Levin and Simon, 1998, Mansvelder et al., 2006). Nicotinic receptors might thus contribute to attention-induced activity changes in higher cortical areas, given that cell-type-specific expression of muscarinic and nicotinic receptors changes with cortical hierarchy (Disney et al., 2006, Disney et al., 2014, Disney and Aoki, 2008). Indeed, in macaque frontal eye field (FEF) muscarinic and nicotinic receptors contribute to neuronal signatures of attention in a cell-type-dependent manner (Thiele et al., 2015). For example, muscarinic receptors contribute to attentional modulation in broad spiking (putative pyramidal) cells, whereas muscarinic and nicotinic receptors contribute to attentional modulation in narrow spiking (putative inhibitory) cells (Thiele et al., 2015).

Together, these data demonstrate that cholinergic signals are involved in attentional signaling, through both phasic and tonic activity. In addition cholinergic input is essential for spatial working memory functions signals in macaque dorsolateral prefrontal cortex (dlPFC). Spatial working memory is severely impaired following cholinergic depletion of the dlPFC (Croxson et al., 2011). Moreover, nicotinic alpha-7 receptor activation is required to enable NMDA-receptor-mediated spatial working memory signals in layer II dlPFC neurons (Yang et al., 2013), whereas nicotinic alpha4beta2 receptors help to maintain spatial working memory signals in dlPFC under distracting conditions (Sun et al., 2017). Based on this, we speculate that nicotinic receptors are also involved in the ability to keep top-down goals in mind (a form of working memory), which may thus affect the strength of attentional control. It is worth noting that the role played by ACh in dlPFC may differ in important ways from its role in V1. In V1, presynaptic NMDA receptors could mediate ACh release to enhance attention, whereas in dlPFC postsynaptic α7 activation supports NMDA-receptor-mediated working memory signals.

### Cholinergic Contribution to Neuronal Coding

At the cellular and network level the effects of ACh mirror those of top-down attention. Attention generally increases neuronal firing rates and most studies report the same when ACh is applied (Disney et al., 2007, Herrero et al., 2008, Pinto et al., 2013, Roberts et al., 2005, Sillito and Murphy, 1987, Thiele et al., 2012, Zinke et al., 2006). Whether the increased activity with attention is a consequence of increased cholinergic drive on a trial-by-trial basis is unknown. Cholinergic transients do not occur on all trials (Sarter et al., 2014). In addition, cholinergic transients have been linked to reward signaling rather than attention (Hangya et al., 2015). This makes a scenario whereby alterations of firing rate on different trials are mediated by alterations in cholinergic drive somewhat unlikely. At the same time, spatial and temporal control of ACh release could in theory be locally induced, even if cholinergic neurons do not change their firing rates (Figure 2B). How could this occur? Transmitter release can be triggered locally within cortical networks through presynaptic NMDA receptor activation, even if the transmitter releasing terminal does not receive (generate) an action potential (Kunz et al., 2013). Additionally, local glutamate release is a requirement for ACh transients in rat frontal cortex (Parikh et al., 2008). It could be this glutamate release that acts on presynaptic NMDA receptors located on cholinergic terminals, whereby ACh is locally released in a temporally and spatially precise manner. Although speculative, such a scenario could explain why attentional modulation of firing rates and firing rate reliability in V1 depends on NMDA receptor availability (Herrero et al., 2013, Self et al., 2012). The general explanation of these results is that the attentional phenomenon seen in V1 are a direct effect of feedback glutamate signaling, which terminates on NMDA rich synapses. However, it is also possible that feedback-induced glutamate release results in NMDA receptor activation on ACh terminals, causing locally increased ACh levels, which increase firing rates and reduce rate variability (Figure 2B for an illustration of the possibility). At least in primate FEF, ACh reduces rate variability in a manner analogous to attention (Thiele et al., 2015, Thiele et al., 2016). So how does increased ACh affect rate variability? We contend this is most likely achieved by overall increased, but balanced, excitation and inhibition. This results in gain changes and stabilized attractor dynamics, which in turn reduce rate variability. Stabilized attractor dynamics in this way have additional benefits. Stable attractor configurations (e.g., directions of attention) are less prone to external perturbation (distractions) and may help task focus over short timescales (Figure 1D). Over longer timescales stabilized attractors could improve overall task focus across trials, conceptualized as “reduced utility cost” (Sarter et al., 2014).

Stabilized attractor networks have additional consequences, which we turn to next. These include altered neuronal correlations and oscillations, which in turn affect the abilities of neuronal populations to encode information and communicate efficiently.

### Cholinergic Effects on Population Activity

Attention and ACh both affect neural population coding abilities. Attention induces desynchronized brain states, which are beneficial for coding (Engel et al., 2016, Harris and Thiele, 2011), it alters oscillatory activity in different frequency bands assumed to aid coding and communication (Chalk et al., 2010, Fries et al., 2001, Gregoriou et al., 2009), and it reduces neuronal co-variability (noise correlations, Cohen and Maunsell, 2009, Herrero et al., 2013, Mitchell et al., 2009, Rabinowitz et al., 2015, Ruff and Cohen, 2014) in ways that should benefit information encoding (Abbott and Dayan, 1999, Panzeri et al., 1999). Whether ACh modulation is causally linked to these attentional effects is largely to be determined (but see Bauer et al., 2012). What has been shown, however, is that increasing ACh in the cortex induces effects that are very similar, if not identical, to those described for attention.

First, ACh is critically involved in altering brain states (Harris and Thiele, 2011, Metherate et al., 1992) (but see Constantinople and Bruno, 2011). Second, ACh can increase stimulus-induced gamma oscillations in visual and prefrontal cortex (Howe et al., 2017, Munk et al., 1996), whereas blockade of muscarinic receptors results in increased low-frequency (<12 Hz) oscillations (Harris and Thiele, 2011). Moreover, increasing cholinergic availability results in an enhanced effect of attention on the hemispheric lateralization of low-frequency oscillations (alpha/beta frequency, Bauer et al., 2012). Third, increasing cortical ACh levels reduces noise correlations (Minces et al., 2017, Thiele et al., 2012). To improve population coding abilities, noise correlations need to be altered in specific ways (Abbott and Dayan, 1999, Panzeri et al., 1999). Whether or not a reduction in noise correlation is beneficial to decoding depends on signal correlations. Neuronal pairs with positive signal correlation should reduce their noise correlations, whereas pairs with negative signal correlations should increase their noise correlations. Such changes can be captured by analyzing the slope of the regression between signal and noise correlation (Minces et al., 2017). A reduced slope is associated with better coding abilities in the population. Attention and increased cortical ACh result in such a reduced slope (Minces et al., 2017, Ruff and Cohen, 2014). These results show that ACh alters the covariance structure of cortical networks, thereby improving population coding, rather than simply altering neuronal gain across the cortical mantle. How could an increase in ACh alter the covariance structure of the cortical network in such a way? First, ACh increases overall neuronal gain (Herrero et al., 2008, Thiele et al., 2012), and it increases the efficacy of feedforward connections (Gil et al., 1999). Both of these changes will result in a cortical network that is less affected by slow global activity fluctuation arising from ‘diffuse’ inputs, and thus will drive noise correlations to values closer to zero (irrespective of whether noise correlations are initially negative or positive). Second, ACh reduces intracortical (lateral) synaptic efficacy through presynaptic M2 receptor activation (Hasselmo and Bower, 1992). These lateral connections are dominated by local neurons and by neurons with similar tuning characteristics (Martin, 2002), i.e., neurons with relatively high signal correlations. Reduction of the efficacy of these connections means that neurons have less mutual impact on one another, which likely reduces their noise correlations. Overall, these alterations would induce the specific change that is required to improve coding (Figure 1C).

In future work, it will be important to determine whether any of the described changes to neuronal activity are specific for different modes of BF cholinergic activity. The slow tonic mode has been argued to be related to global and enduring behavioral state changes, whereas the rapidly fluctuating phasic mode with high temporal precision has been linked to reinforcement learning (Hangya et al., 2015), reward (Richardson and DeLong, 1986), and cue detection in an attention task (Parikh et al., 2007). Notably, brain areas such as the orbital PFC and the insular cortex that encode high-level reward and utility have strong projections to the BF (Mesulam and Mufson, 1984), which could supply a reward/utility signal to cholinergic neurons. The resulting cholinergic signal might then set a cortical processing mode whereby task focus (reward exploitation) is favored. One may thus ask whether fluctuating levels of ACh release are involved in the fluctuating allocation of top-down attention. To answer this, additional cyclic voltammetry in different areas and species or 2-photon imaging of cholinergic terminals in various areas would yield invaluable insight. Alternatively, ACh release, in its tonic mode, might simply shape cortical network interactions such that attention can allocate neuronal resources adequately. In the latter scenario, other sources would provide the temporal and spatial specificity (e.g., feedback from higher cortical areas) to induce the fine-grained coding changes seen with different forms of top-down attention. Proposals have been made where alteration in ACh signals directly link to the allocation of attention (Herrero et al., 2008, Sarter et al., 2009, Thiele, 2013). Even if ACh simply acts to enable these behavioral effects, it is crucial that we delineate the exact mechanism, as this might provide a more nuanced perspective on the use of cholinergic agents to enhance attention.

### Attention and the Dopaminergic System

DA is strongly linked to reward signaling and the economic decision variable of utility (Schultz et al., 1997, Schultz et al., 2017, Stauffer et al., 2016) and learning (Waelti et al., 2001). In addition it is a key neuromodulator supporting prefrontal spatial working memory signals (Arnsten et al., 1995, Arnsten et al., 2012, Sawaguchi and Goldman-Rakic, 1991, Wang et al., 2004, Williams and Goldman-Rakic, 1995). The DA system is also a primary pharmacological target for disorders such as attention deficit hyperactivity disorder (ADHD), schizophrenia, and Parkinson’s disease, which are associated with attention deficits (Arnsten and Rubia, 2012).

**Figure 3:**
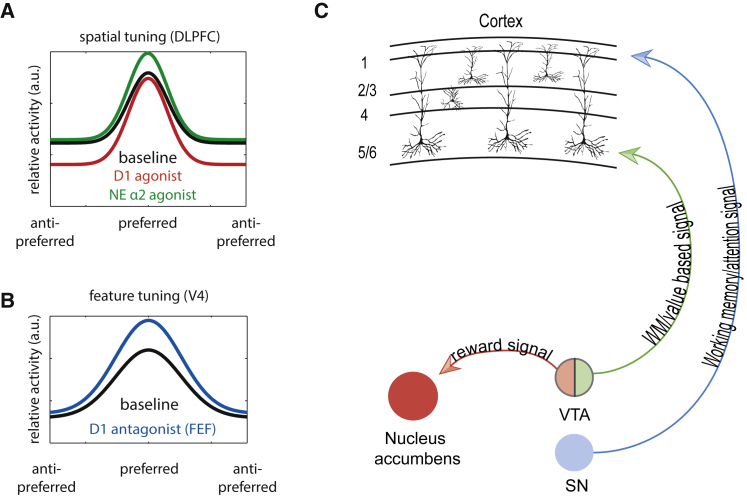
Neuromodulation of WM Fields, Remote Feature Tuning, and Specificity of Dopaminergic Output Signals (A) Spatial tuning of WM fields is enhanced when small amounts of D1 agonists are applied in the vicinity of the neurons, by selectively reducing activity for non-preferred locations (memory fields). This is equivalent to a noise reduction. Spatial tuning of WM fields is equally enhanced when small amounts of NA α2A agonists are applied in the vicinity of the neurons, by selectively increasing activity for preferred locations (memory fields). This is equivalent to signal enhancement. Both changes increase the SNR. (B) Application of D1 antagonists to area FEF enhances the tuning of area V4 neurons that have overlapping receptive fields with the affected FEF locations. (C) Hypothetical interactions of dopaminergic subpopulations carrying specific; based on projections found in rat. dlPFC, dorsolateral prefrontal cortex; FEF, frontal eye field; VTA, ventral tegmental area; SN, substantia nigra; a.u., arbitrary units.

Although long hypothesized, it has recently been shown that DA contributes to spatial attention and to target selection in macaque FEF (Noudoost and Moore, 2011a, Noudoost and Moore, 2011b, Soltani et al., 2013). Noudoost and Moore (2011a) engaged animals in a free saccade target selection task, where target onsets were systematically altered. This led animals to more often choose those targets that appeared earlier. Infusion of D1 antagonists and D2 agonists into selected locations of primate FEF systematically shifted the choice function, biasing choices toward locations represented by neurons that were affected by the DA manipulations. In addition, in a passive fixation task, the infusion of D1 antagonists into FEF altered neuronal responses in remote area V4, as if attention had been allocated to the receptive field of those neurons (Figure 3B; Noudoost and Moore, 2011a). The V4 response parameters affected were firing rate, sharpness of tuning, and rate variability. However, no effects were found on V4 activity, when D2 agonists were infused into FEF. The discrepant results for behavior and neuronal recordings can be explained by layer-dependent expression of D1 and D2 receptors in macaque cortex. D1 receptors are expressed in supragranular layers (which project to area V4) and infragranular layers of the prefrontal cortex, whereas D2 receptors are expressed only in infragranular layers, which provide output to the midbrain and brainstem (Lidow et al., 1991). It may be surprising that a reduction in D1 receptor action in FEF results in increased behavioral choices of targets presented at the location the FEF neurons represent, and in increased activity of V4 that overlaps with the spatial representation of the affected FEF neurons. However, this could result from the mostly inhibitory nature of D1 action. Blocking this would give the affected FEF neurons a competitive advantage for selection compared with other FEF neurons and may thereby trigger winner-take-all states in the absence of a cognitive trigger signal. This would then enhance the feedback signal to V4 in a spatially selective manner. In a follow-up analysis, these authors used neural network analysis to show that D1 receptors affected choices by increasing the efficacy of inputs and recurrent connections, whereas D2 receptors affected choices by increasing output efficacy (Soltani et al., 2013). The ongoing development of selective D1 agents for use in humans (Arnsten et al., 2017) will provide an opportunity in the near future to arbitrate between the relative roles of D1 and D2 receptors in spatial attention. Already in line with the macaque studies, human work failed to identify a modulatory effect of D2 receptor activation on visual perception (Gratton et al., 2017).

In spatial working memory tasks, which are conceptually related to top-down spatial attention, application of a small dose of D1 agonist increases spatial tuning of macaque dlPFC memory fields by reducing the activity for non-preferred locations (Figure 3A; Vijayraghavan et al., 2007), whereas application of a D1 antagonist in dlPFC improves the spatial tuning of memory fields by enhancing preferred location activity (Williams and Goldman-Rakic, 1995). Conversely, D2 agonists do not affect spatial coding during the working memory delay period but increase saccade-related activity for preferred target locations. Placed within the context of attention, this would largely match the results reported by Noudoost and Moore (2011a), where D1 receptors are involved in the generation of top-down attentional signals (assumed to be reflected by activity in selected FEF neurons, which affect sensory areas), whereas D2 receptors, located in output layer 5, are involved in motor-related choice activity (Noudoost and Moore, 2011a, Soltani et al., 2013). The functional dissociation between D1 and D2 receptors and their layer-dependent expression raises the question of whether DA inputs are dissociable. From an anatomical perspective, work in rodents provides evidence in favor of this view. The two main sources of cortical DA, namely, the ventral tegmental area (VTA) and the substantia nigra (SN) have somewhat different termination patterns in PFC. Input arising from the rodent SN preferentially terminates in layer I, whereas input from the VTA preferentially terminates in layer V (schematic in Figure 3C; Berger et al., 1991). Interestingly, layer 5 inputs, which are modulated by D2 activation, are assumed to carry value-based information (in a model of macaque FEF; Soltani et al., 2013), whereas inputs to superficial layers, modulated by D1 activation are assumed to carry stimulus-related information (Soltani et al., 2013). Speculatively, then SN-DA signals might be more task and attention related, whereas VTA-DA signals might be more reward related. Indeed, such differences have been observed in working memory tasks (Matsumoto and Takada, 2013). Whether these results hold in a top-down attention task needs to be determined. However, in rodents performing a 5 choice serial reaction time task (5-CSRTT) it was found that increasing the activity of midbrain dopaminergic neurons using chemogenetics was detrimental to attentional performance, but not response inhibition (Boekhoudt et al., 2017). Importantly, different aspects of attention deteriorated when VTA- or SN-DA neurons were affected. Increasing VTA- or SN-DA activity caused increased numbers of trial omissions, whereas accuracy (a measure of attentional performance; Bari et al., 2008) was only affected when SN-DA neuronal activity was increased (Boekhoudt et al., 2017). Finally, behavioral features common to attentional dysfunctions, such as hyperactivity, can be induced by increasing the activity of VTA- not SN-DA neurons. This hyperactivity was induced when increasing the activity of VTA-DA neurons projecting to the nucleus accumbens, but not of those projecting to prefrontal cortex (Boekhoudt et al., 2016). Taken together, these findings are in line with the hypothesis that DA signals arising in the SN and terminating in superficial PFC aid the generation of feedback attention signals, whereas those arising in VTA and terminating in infragranular layers are related to reward/reward prediction error signaling and thus regulate choice signals (Figure 3C for a schematic thereof). Additionally, specific subsets of VTA-DA neurons in rodents are involved in either working-memory-related or reward-related activity and have different output and input connectivity. Those involved in working memory (WM) project preferentially to the PFC, whereas those involved in reward/motivation signaling project preferentially to the nucleus accumbens and orbitofrontal cortex (OFC) (Lammel et al., 2008). Finally, optogenetic activation of their respective input (lateral habenula versus laterodorsal tegmentum) elicits aversion- versus reward-related behaviors (Lammel et al., 2012, Lammel et al., 2014). Whether similar distinctions exist in primates is unclear and additional work in rodents is required to test the hypothesized relationships between these anatomically segregated connections and the specific signals they might carry. Regardless, it is now well established that DA signals have a variety of different effects on cortical networks, and importantly they serve different functions. These are, at least in part, segregated into specific anatomical subdivisions. Together these multiple effects of DA on cognitive functions helps to strengthen specific aspects of mental representations (Arnsten, 2011), for example, sculpting representation of sensory inputs, and biasing outputs in favor of choices that have a good history of yielding rewards. Within this context, it may not matter whether the mental representation is rule based, WM related, or a top-down attention monitoring signal. It seems clear that DA contributes to all of these, and all of these mental operations are impaired in psychiatric and neurological disorders of putative dopaminergic origin. Given the illness burden associated with these disorders, there is a clinical imperative for the ongoing development of more selective DA agents. Significant advances have been made in this space with the development of selective D1 agonists (e.g., dihydrexidine), which offer the promise of remediating WM and attention deficits in disorders such as schizophrenia (Arnsten et al., 2017).

### Attention and the Noradrenergic System

The noradrenergic (NA) system affecting the cortex arises from neurons located in the locus coeruleus (LC, Loizou, 1969). Theorizing around the function of the noradrenergic system has paralleled that of the other neuromodulatory systems, being argued to be non-specific in its projection and in its function. It was assumed that the cortical projection had very limited anatomical specificity, other than possibly at the level of right hemisphere dominance in humans (Grefkes et al., 2010). Moreover, the assumption was that the level of activity in NA neurons, which varied between different sleep and wake states determined the level of “arousal” (for review, see, e.g., Aston-Jones and Cohen, 2005, Aston-Jones and Waterhouse, 2016). Neurons fire tonically at low rates during sleep, at higher rates during normal wake states, and at yet higher rates when under stress (Aston-Jones and Bloom, 1981, Aston-Jones et al., 1991, Foote et al., 1980). These different rates of activity can be linked to differences in the ability to perform cognitively demanding tasks, with low performance when tonic activity is either low or very high (Aston-Jones and Cohen, 2005, Aston-Jones et al., 1999).

Formulating a coherent theory around NA functions is complicated by that fact that NA has different and sometimes opposite effects dependent on the circuit examined. For example, in sensory cortex NA affects its target neurons through α1, α2, and β adrenergic receptors. α1 receptor activation generally causes excitation, α2 activation causes inhibition (reviewed in Berridge and Waterhouse, 2003), and β adrenergic activation generally increases excitability (McCormick et al., 1991). The overall effect of NA release at the cellular level is a reduction in spontaneous activity and an increase in input-driven activity provided the stimulus is salient (or preferred), thereby improving the SNR (in a manner similar to the filter gain change in Figure 1A; Foote et al., 1980, Waterhouse et al., 1988, Waterhouse et al., 1998). This specific behavior sets it apart from the action of, e.g., ACh or DA, which, if excitatory, result in either no change of spontaneous activity, or even mild increases of spontaneous activity (i.e., akin to the “response gain” change illustrated in Figure 1A). At the single-cell level, NA might thus cause a shift toward saliency detection, where weak stimuli are filtered out, and salient stimuli elicit strong responses (Figure 1A; for an example, Servan-Schreiber et al., 1990). ACh has been hypothesized to cause similar action, not necessarily by altering single-cell filter gain mechanisms uniformly, but rather by network reconfiguration (Thiele, 2013). However, in primate dlPFC α2 receptor stimulation of layer III increases delay cell firing (Li et al., 1999) and reduces distractibility (Arnsten and Contant, 1992), while α1 receptor stimulation decreases delay cell firing (Arnsten, 2011). This discrepancy between the effects of NA on sensory cortex and PFC suggests that NA in PFC may be involved in top-down control (attention, WM) through α2 activation, whereas it is involved in bottom-up state control through α1 activation in sensory cortex. These control mechanisms would be differentially activated based on levels of NA release, as α2 and α1 receptors have differential NA affinity.

As stated previously, one of the main arguments against implicating neuromodulators in top-down attentional signaling is the fact that top-down attention is highly specific in the spatial and/or the feature domain, which affects very localized (or at least very specific) neuronal populations. Although the spatial specificity of the NA projections are insufficient to yield the required top-down resolution, the NA system nevertheless mediates the spatial specificity of WM signals, by dynamically sculpting local network interactions (reviewed in Arnsten et al., 2012). Moreover, the NA projection system is “less unspecific” than previously argued. For example, certain subsets of NA neurons exclusively project to the insular cortex, where they aid in the analysis of enteroception, and these NA neurons in turn are affected by afferents from the enteroceptive system (Chandler et al., 2014a, Waterhouse and Chandler, 2016). Moreover, a subset of LC neurons project only to the PFC and not to motor cortex in the rat (Chandler et al., 2014a). LC projections to the PFC differ from those to the orbitofrontal cortex. The former support attention and extradimensional shifting, whereas the latter support reversal learning (Chandler et al., 2014b). Finally, specific prefrontal neurons, even if localized in the immediate vicinity of one another, are differently affected by neuromodulation, such that layer 5 cells, which project to the brainstem, show different general response properties and are affected differently by NA activation (through α2A receptor activation), than intracortically projecting neurons (Dembrow and Johnston, 2014, Dembrow et al., 2010). These results are testament that the brainstem neuromodulator system, even if comparatively unspecific, can mediate very specific and localized effects at cortical target sites.

As is the case for other neuromodulators, NA neurons also engage in a phasic response mode, where they respond to behaviorally relevant stimuli with brief bursts of activity (Aston-Jones et al., 1991, Aston-Jones et al., 1994, Aston-Jones et al., 1997, Clayton et al., 2004). Given that the phasic activity is task dependent (larger when the animal performs the task well, and larger on correct than on error trials), and it temporally precedes the behavior, it has been suggested that it provides a “temporal attention filter, that facilitates task relevant behaviour” (Aston-Jones and Cohen, 2005). Alterations in baseline (tonic) activity are also linked to behavior, but in a very different manner. Higher baseline NA activity is accompanied by reduced task performance and increased distractibility (Rajkowski et al., 1994). Based on this discrepancy, it has been argued that strong phasic task-related activity (in conjunction with low tonic LC-NA activity) helps the animal to stay task focused (and thereby exploit current reward contingencies; “exploitation”), whereas the high tonic activity (in conjunction with low phasic LC-NA activity) supports a mode where task focus is low and alternative behaviors are explored for their potential benefits (“exploration”, Aston-Jones and Cohen, 2005, Usher et al., 1999; for an interpretation in terms of uncertainty resolution, see also Yu and Dayan, 2005). This distinction can also be couched in terms of high versus low neural gain, which is increasingly thought to influence attention and decision making (Eldar et al., 2013). In the context of attractor networks, the former would be associated with a stabilized state, while the latter would be associated with a less stable state.

Which of the two NA modes directly affect PFC-dependent cognitive signals is not entirely clear, but the tonic mode is a likely candidate. The role of NA on cognitive signals in the PFC has best been studied in relation to spatial WM (Arnsten, 2011, Arnsten, 2013, Arnsten and Jin, 2014, Robbins and Arnsten, 2009). At moderate (non-stressed) levels, NA improves WM performance through α2A receptor activation (Arnsten and Goldman-Rakic, 1985). High levels of NA impair PFC activity and WM performance through stimulation of α1 (Mao et al., 1999) and β1 receptors (Ramos et al., 2005), which have lower NA affinity. The WM delay activity in the prefrontal cortex, while mediated through recurrent excitation dependent on NMDA receptor activation (Wang et al., 2013), is strongly modulated by α2A receptor activation (Wang et al., 2007). The α2A receptor activation improves spatial tuning of WM-related delay activity in PFC neurons, by increasing preferred spatial memory locations, without affecting non-preferred locations (see Figure 3A for a schematic). Although a number of studies in humans have also manipulated α2A signaling during tasks of spatial attention, results are inconclusive with some studies reporting effects of clonidine (Coull et al., 2001), but not others (Gratton et al., 2017).

A role for NA in other aspects of top-down attention, such as sustained attention, is, however, supported. Low-dose clonidine, which acts pre-synaptically to reduce NA cell firing and release, increases attentional lapses (Smith and Nutt, 1996). Notably, this effect was reversed by treatment of a selective alpha-2-adrenoceptor antagonist. Moreover, methylphenidate, a psychostimulant used in the treatment of ADHD, which enhances NA and DA signaling and improves sustained attention (Dockree et al., 2017). It is plausible that this effect is at least partially attributable to modulation of α2A receptors, since methylphenidate robustly modulates these receptors in PFC.

In summary, multiple lines of evidence support the view that the LC-NA system exerts an important neuromodulatory influence on attention. Although animal work shows potent modulations of cells and circuits supporting spatial WM and attention by NA agents, identifying specific cognitive effects of these agents in humans has been a challenge. Part of the challenge in human work results from the sedating effects of acute dosing of α2A agents (e.g., clonidine, guanfacine). This yields noisy behavioral performance and difficulty in discriminating between task-specific effects of the drug and the non-specific effects of sedation. Although sedation wanes with chronic dosing, such regimens are ethically hard to justify in the non-clinical cohorts that are often employed in psychopharmacology. Establishing specific effects of α2A agents on top-down attention is therefore likely to require the recruitment of clinical cohorts with a clinical indication (e.g., guanfacine in ADHD).

### Attention and the Serotonergic System

The contribution of 5-HT to top-down attention may be less direct than that described for ACh, DA, and NA. However, varying levels of 5-HT do affect the ability to engage and perform well in top-down attention and spatial WM tasks.

For example, 5-HT affects spatial tuning of putative pyramidal cells in a memory-guided saccade task in macaque dlPFC. Blockade of 5-HT_2A_ receptors resulted in reduced spatial tuning, whereas activation of these receptors caused increases in spatial tuning by either increasing activity for preferred target locations, and/or reducing activity for non-preferred target locations (Williams et al., 2002). Given the proposed link between spatial WM and spatial attention, we would speculate that similar effects will be found in top-down attention tasks in dlPFC (and possibly the FEF). However, it is equally possible that very different effects occur when using an attention task, as the 5-HT _1A/2A_ agonist psilocybin impairs attentional tracking in humans, without affecting spatial WM. The former was interpreted as resulting from reduced ability to suppress distracting stimuli, rather than reduced attentional capacity per se (Carter et al., 2005).

Reduced attentional performance is also seen under other conditions. Systemic injection of a 5-HT_2A_ agonist in rats results in reduced accuracy (attention) and increased impulsivity (response disinhibition) in 5-CSRTT (Koskinen et al., 2000). However, direct infusion of a 5-HT_2A/C_ antagonist into rodent mPFC only reduced impulsivity, without affecting attention (Passetti et al., 2003). This discrepancy could indicate that effects on attention/accuracy are induced by 5-HT action in areas different from mPFC. Blockage of 5-HT_1A_ and 5-HT_2A_ receptors offsets the 5-CSRTT performance deficits seen when NMDA receptors are blocked (Carli et al., 2006, Ceglia et al., 2004). Despite this common effect on accuracy overall, the two receptor subtypes have dissociable functions in relation to attention (accuracy). 5-HT_1A_ blockade improves accuracy by reducing NMDA receptor blockade-induced perseverance, whereas 5-HT_2A_ blockade affected accuracy by reducing impulsivity. Based on this dissociation, it has been suggested that 5-HT_2A_ receptors are critical to modulate attentional control of response inhibition (Aznar and Hervig, 2016).

A role of 5-HT in attentional processes has also been demonstrated in a reversal learning task. Reversal learning describes the phenomenon where subjects have to inhibit responses that are no longer rewarding, shift attention to alternative stimuli, which could be associated with reward, and evaluate the risk/benefit in responding to these novel stimuli. It requires cognitive flexibility and is often assessed using the Wisconsin card sorting test in human and non-human primates (Grant and Berg, 1948, Nakahara et al., 2002), or the attentional set shifting task in rodents (Kesner and Churchwell, 2011). The main anatomical area involved in reversal learning is the orbitofrontal cortex, with its projections to the medial and ventral striatum. Reversal learning requires adequate 5-HT levels, which helps updating of the value/utility of responding to novel (not previously rewarding) stimuli and allows inhibition of previously rewarded responses (Roberts, 2011). In addition to the orbitofrontal cortex, rodent mPFC (or primate dlPFC) are also involved in reversal learning, when extra-dimensional shifts or new stimulus reward association learning (a shift in attention) is required. Extradimensional shifting is improved by 5-HT_2A_ receptor blockade (Passetti et al., 2003), or generally reduced 5-HT input to mPFC (dlPFC). Thus, 5-HT_2A_ receptors in mPFC/dlPFC would be involved in attentional control, whereas 5-HT_2A_ receptors in orbitofrontal cortex are involved in cognitive flexibility and reversal learning (Aznar and Hervig, 2016). Attentional control would, however, be affected by 5-HT_2A_ in an indirect (or negative) manner, whereby low levels of 5-HT improve attention. Unsurprisingly, 5-HT depletion results in improved attentional control in humans (Scholes et al., 2007). Activation of 5-HT receptors results in increased distractibility (associated with reduced focus on recently rewarded items or behaviors) and in increased cognitive flexibility when reward contingencies have changed (Baker et al., 2011, Terry et al., 2005). This is reminiscent of the exploitation versus exploration functions that have been discussed in relation to NA, but by different mechanisms. Exploitation would be governed by low tonic 5-HT in mPFC and orbitofrontal cortex, whereas exploration would be induced when 5-HT levels are high in either area. Potential cellular mechanisms for this effect have been described by Tian et al. (2016). These authors report that 5-HT inhibits mouse layer 6 mPFC pyramidal cells through 5-HT_1A_ and 5-HT_2A_ receptors. This inhibition results in reduced activity in layer 5 interneurons, thereby increasing what the authors define as “noise” in the layer 5 output layers. A consequence thereof could be an overall reduced threshold to engage in “untested” exploratory behaviors, i.e., a form of exploration.

Overall, the above may fit the notion that 5-HT neurons in the dorsal raphe nucleus contain information of the “state value” or “reward value” of the current situation (Roberts, 2011). However, the neurons in the dorsal raphe nucleus encode positive as well as aversive future outputs (Bromberg-Martin et al., 2010, Li et al., 2016, Miyazaki et al., 2014), and these can even involve highly specific pathways (Marcinkiewcz et al., 2016). Thus, the coding of dorsal raphe 5-HT neurons appears more diverse than simply representing “reward value.”

### Summary and Outlook

Here, we have reviewed the critical role played by neuromodulators (ACh, DA, NA, and 5HT) in mediating attention-induced modulations of neuronal activity at the single-neuron and circuit level. Similarities of action exist across these neuromodulators, such as the common existence of phasic versus tonic modes, with effects that follow U-shaped dose-response relationships, whereby too little or too much neuromodulator drive is detrimental to cognition. However, important differences between their actions also exist.

#### ACh

Arguably, the classical view is that neuromodulation of attention occurs via cholinergic mechanisms. Yet as we have reviewed, significant gaps in our knowledge exist regarding the specific roles of receptor and cell subtypes, release mechanisms, and local control thereof. At the single-neuron level, ACh mediates attention-induced rate changes that vary regarding their specific receptor involvement between lower (e.g., V1) and higher cortical areas (e.g., FEF). It will be important to delineate these differences in more detail for different excitatory and inhibitory cell types, at different levels of the cortical hierarchy. At the circuit level, ACh reduces rate variability and co-variability via enhanced gain and stabilized attractor dynamics, thereby improving population coding abilities. Behaviorally, stabilized attractors may reduce moment-to-moment distractibility as well as promoting longer-term task engagement.

#### DA

A role for DA in top-down spatial attention is supported by animal work, but ultimately receptor- and cell-type specificity requires further clarification. For example, the relative expression of D1 and D2 receptors in supragranular (derived from SN-DA neurons) versus infragranular (derived predominantly from VTA-DA neurons) layers of FEF is important for attention. The supragranular layers send feedback to V4 and passive infusion of D1, but not D2, agonists into FEF engendered attention-like modulations of V4 activity (e.g., increased firing rates; reduced rate variability). However, how D1 activation/blockade shapes attention feedback signals at the local level in FEF is currently unknown, and it is unclear whether these effects differ for tonic versus phasic release modes. Overall, these and other data support a view that DA signals arising from SN and terminating in supragranular layers of FEF allow specific local networks to strengthen feedback attention signals. Conversely, those arising from VTA and terminating in the infragranular layer of FEF are related to reward/prediction error signals and directly bias choice signals without necessarily affecting upstream processing.

#### NA

Despite the diffuse projection of the NA system to the cortex, NA influences a range of discrete cognitive processes (e.g., spatial specificity of WM signals) mediated by specificity in both the receptor subtypes and projections of the NA system. An overall increase in NA release at the cellular level decreases spontaneous activity and increases input-driven activity to salient (behaviorally relevant) stimuli, thereby enhancing signal. NA release modes also vary, whereby a low tonic, high phasic release enables animals to stay task focused and exploit existing (predictable) reward contingencies. How phasic release affects sensory processing, or attentional (feedback) and motor/choice planning (feedforward) signals in cortical areas will be an important area for future studies.

#### 5-HT

Although the role of 5-HT in attention has been less intensively studied, varying levels of 5-HT do influence top-down attention and spatial WM. Some evidence for receptor level dissociation of function exits, with 5-HT_1A_ blockade improving accuracy by reducing NMDA receptor blockade-induced perseverance, whereas 5-HT_2A_ blockade affects accuracy by reducing impulsivity. Expression of 5-HT_2A_ in mPFC/dlPFC appears involved in attentional control, whereas 5-HT_2A_ receptors in orbitofrontal cortex are involved in cognitive flexibility and reversal learning. Current evidence suggest that attentional control is affected by 5-HT_2A_ in an indirect (or negative) manner, whereby low levels of 5-HT would improve attention, by promoting task focus, whereas high 5-HT levels support disengagement.

Overall, this review highlights much greater functional and anatomical specialization in subcortical neuromodulator systems than previously argued. Neuronal subpopulations of the different neuromodulators appear to control separable aspects of attention. Their main role is to allow for enhanced flexibility of cortical coding. Thereby these neuromodulators configure how (and which) specific cortical areas affect behavior. This added flexibility and specificity is recapitulated within cortical layers of an area (e.g., D1 supragranular versus D2 infragranular layers within FEF). Further, as described for NA, specificity of receptor expression and thus neuromodulator influences even exist at the level of local layer 5 pyramidal cells, provided they differ in their output connectivity. Novel genetic tools that allow the tracing and interrogation of specific sub-circuits within these neuromodulator systems have provided invaluable insights at the single-cell, population, and behavioral level. These tools have mostly been employed in rodents, as cell-type-specific targeting is more advanced in these species than currently possible in non-human primates. However, progress is also being made in non-human primates to advance these tools. These advances should ultimately inform our understanding of human attention. Developing these techniques in non-human primates will be necessary because cell-type-specific expression of neuromodulator receptors can differ radically between rodents and primates (Disney and Reynolds, 2014). Innervation pattern can also differ. For example, the dopaminergic innervation of “attention areas” such as the parietal cortex differs radically between rodents and primates (Berger et al., 1991). Critically, the absence of a dlPFC homolog in rodents—a key area controlling attention and WM in primates—(Preuss, 1995) is yet another strong argument for the necessity of non-human primate research.

Important future insights into the mechanistic roles of neuromodulators can also be obtained from modeling studies (Dayan, 2012). Many of the models involved are based on architectures, which exhibit the described attractor dynamics and winner-take-all characteristics and have been successfully implemented, for example, to disentangle the mechanistic roles of muscarinic and nicotinic receptors on attentional bias in macaque V1 (Deco and Thiele, 2011).

Although the field eagerly awaits these advances, it is clear that the extant data refute the prevailing classical view that brainstem neuromodulator systems simply modulate global brain states. Rather, the emerging view is one in which receptor and cell-subtype-specific effects of neuromodulators influence the efficiency of large- and small-scale neuronal networks to instantiate the top-down control of attention.
